# Concurrent Xanthogranulomatous Pyelonephritis and Upper Urinary Tract Transitional Cell Carcinoma

**DOI:** 10.1155/2023/6021178

**Published:** 2023-03-31

**Authors:** Anthony Guglin, Robert Weiss, Adityabikram Singh, Anugya Mittal, Thomas Hwang, Ankit Shah

**Affiliations:** ^1^Department of Surgery-Urology Division, Rutgers New Jersey Medical School, Newark NJ, USA; ^2^Department of Medicine-Hematology and Oncology Division, Rutgers New Jersey Medical School, Newark NJ, USA; ^3^Rutgers Cancer Institute of New Jersey, Newark NJ, USA

## Abstract

A 37-year-old male with a history of chronic nephrolithiasis presented to the ED with gross hematuria, clot retention, and right flank pain. The patient had radiological findings of perinephric stranding, marked hydronephrosis, and marked thinning of the right renal parenchyma on computed tomography (CT), all suggestive of xanthogranulomatous pyelonephritis (XGP). The specimen following radical nephrectomy revealed urothelial carcinoma (UC) in a background of XGP but with no evidence of spread to regional lymph nodes. Follow-up imaging revealed hypodense lesions in the liver which demonstrated UC on biopsy. This is the first reported case of a young patient presenting with such an advanced stage of UC in the setting of XGP. It illustrates the link between inflammatory processes of the kidney and malignancy of the upper urinary tract.

## 1. Introduction

Xanthogranulomatous pyelonephritis (XGP) and upper tract urothelial carcinoma (UTUC) are independently rare diseases [1, 2]. This report describes a 37-year-old male who was initially diagnosed with XGP, and following resection, pathology showed the presence of UTUC in a background of XGP. There have been few cases reported of concurrent XGP and UTUC [3, 4], but none of the reported patients presented at an advanced stage of disease at such a young age.

Xanthogranulomatous pyelonephritis is a rare form of a granulomatous reaction to chronic pyelonephritis. Patients with XGP develop severe kidney damage because of the destructive granulomatous process affecting the renal parenchyma [5]. The disease process often stems from long-term obstruction and infection, most commonly involving *Escherichia coli* or *Proteus mirabilis* [2]. However, there has also been a case reported of focal XGP with no history of obstruction [6]. XGP accounts for 0.6% to 1% of all cases of pyelonephritis, and there are 1.4 cases per 100,000 people each year [2, 7–9]. The mean age at diagnosis is 45-55.2 years, and women are more frequently affected than men [2, 7–9]. Almost all patients are symptomatic, and the most common symptoms are flank or abdominal pain, lower urinary tract symptoms, fever, palpable mass, gross hematuria, and weight loss [2]. The initial diagnosis is made via CT imaging with confirmation via gross pathologic and microscopic exam of nephrectomy specimen. XGP may present as an infiltrative renal cell carcinoma [6, 10]. The two forms of XGP are diffuse and focal, and radical nephrectomy is the indicated treatment for diffuse or advanced-stage disease. The inflammatory process of XGP can frequently involve or extend beyond Gerota's fascia, often encompassing nearby structures and requiring further resection. Nevertheless, XGP is typically a unilateral disease, and prognosis is favorable following timely treatment [7].

Urothelial carcinoma (UC) is a malignancy arising from the transitional cells of the urinary tract system. UC most commonly occurs in the bladder while a minority, 5-10%, of urothelial tumors are found in the upper urinary tract [1]. The estimated incidence of UTUC is 1 to 2 cases per 100,000 persons per year [11]. Smoking is the most important risk factor while exposure to occupational carcinogens, heavy coffee consumption, and cyclophosphamide are also often implicated in the etiology. Gross or microscopic hematuria is the most common presenting symptom and appears in 70-80% of patients [12]. Less common symptoms include flank pain, mass effect, urinary frequency, and weight loss. CT has the highest diagnostic accuracy of all imaging techniques [13]. The sensitivity of CT urography for UTUC is 92% with a specificity of 95% [13]. Management depends on the disease severity but can include nephroureterectomy, endoscopic resection, or chemoablative therapies for local disease and chemotherapy/immunotherapy for metastatic disease [14]. Herein, we discuss a case of concurrent xanthogranulomatous pyelonephritis and upper urinary tract transitional cell carcinoma.

## 2. Case Presentation

**Figure 1:**
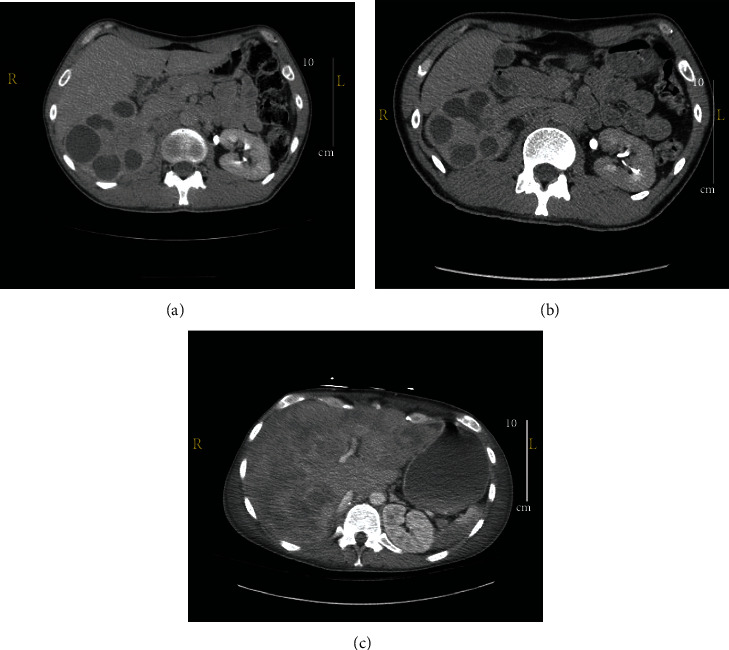
Radiographic findings. (a) Contrast-enhanced CT abdomen on initial presentation demonstrating an enlarged right kidney with hydronephrosis and perinephric stranding which is consistent with the “bear claw sign” that suggests XGP. (b) Contrast-enhanced CT abdomen prior to nephrectomy demonstrating aggressive progression of right kidney disease with invasion of surrounding structures. (c) Contrast-enhanced CT abdomen demonstrating multiple biopsy-confirmed metastatic liver lesions from urothelial carcinoma.

A 37-year-old male with a medical history of nephrolithiasis, latent tuberculosis, and a 20+ pack-year smoking history initially presented in February 2021 with complaints of hematuria, right flank pain, and new onset clot passage. A renal and bladder ultrasound was then done which showed numerous irregular cysts in the right kidney, a normal left kidney, a heterogeneous mass in the bladder, and clot passage. A cystoscopy was performed which visualized the clot but detected no cancerous lesions or stones. Cytology from the bladder washing was negative. Urine cultures and an acid-fast bacteria stain were negative. A CT chest, abdomen, and pelvis with contrast was performed which demonstrated perinephric stranding, marked hydronephrosis, and marked thinning of the right renal parenchyma (Figure 1(a)). A nuclear medicine renal scan indicated impaired right kidney function (33%) with preserved left kidney function (67%). These findings were consistent with right-sided XGP, but a formal diagnosis could not be made without the pathology of a specimen. Given the normal creatinine and preserved left renal function, a simple right nephrectomy was recommended. The patient declined the operation and was discharged against medical advice.

He returned to the ER two months later with similar complaints as before of right flank pain, dysuria, and hematuria. He was able to find relief with over-the-counter nonsteroidal anti-inflammatory drugs and agreed to undergo a nephrectomy. A CT abdomen/pelvis showed significant progression of the right XGP as well as a new infiltrative process of the right renal pelvis with possible right renal vein thrombosis (Figure 1(b)). The patient underwent an open right radical nephrectomy to treat the extensive XGP during this hospital admission. Pathology of the specimen showed UC with squamous differentiation in a background of XGP. Specimen margins were negative for invasive carcinoma without evidence of tumor in regional lymph nodes. A CT abdomen/pelvis two months later in September 2021, however, was significant for multiple new poorly defined hepatic hypodensities with concern for metastatic disease versus abscesses related to disseminated infection (Figure 1(c)). The patient also later endorsed the onset of bone pain.

CT-guided liver biopsy was ordered which confirmed metastatic UC. However, nuclear medicine bone scan and CT chest with contrast were negative for osseous metastasis and thoracic lesions or lymphadenopathy, respectively. The patient was then evaluated by medical oncology and was started on gemcitabine and cisplatin (GC) palliative chemotherapy given the progression to metastatic disease.

Within one week of chemotherapy induction, the patient required hospital admission to treat sepsis secondary to mucositis. Following discharge, he was able to resume his chemotherapy regimen. A CT chest, abdomen, and pelvis performed after the third cycle of GC demonstrated interval reduction in the size of liver lesions without any appreciable change in abdominal lymphadenopathy. Given the patient's ongoing clinical response, the decision was made to complete 6 cycles of platinum-based chemotherapy. He was eventually treated with avelumab maintenance. Despite this, the patient had rapid disease progression. Given his overall critical status and poor prognosis, the decision was made to transition patient to hospice, and he eventually passed away due to the disease.

## 3. Discussion

This patient's case is unique in that he presented with a rare case of concurrent XGP and UTUC. Both pathologies are independently very rare diseases [1, 2], so to see them present together raises suspicion that one process may affect the development of the other. Although there is little literature discussing XGP and UTUC, a 2001 retrospective study revealed that one of ten patients with XGP developed UC of the renal pelvis [8]. This study's hypothesized mechanism of the association is that the development of UC initiates a xanthogranulomatous response. Our patient was first diagnosed with XGP by radiographic findings while UTUC was diagnosed only after histological analysis of the nephrectomy specimen. A plausible hypothesis is that the patient first developed XGP and then UTUC in response to the chronic inflammation seen with XGP. Researchers have recently correlated chronic inflammation and variations in the bladder microbiome with the development of bladder cancer [15]. It is conceivable that a similar mechanism exists for upper tract tumors given the similarities in epithelial lining and microbiome between the urinary bladder and renal pelvis.

It is important to note that radiographic findings of XGP cannot exclude other diagnoses. XGP is diagnostically a “great imitator” as the imaging findings parallel those found in other disease processes including infection and malignancy. Given the patient's history of heavy tobacco use and latent TB, differentials such as renal cell carcinoma, UC, and tuberculosis remain important considerations [15]. The characteristics of CT findings of XGP include dilated calyces, changes in renal size and shape, and the moderately specific but not pathognomonic “bear claw” sign. The bear claw sign, as seen in this patient, describes the appearance of multiple round regions of low-attenuation signals (-10 to +30 Hounsfield units) radiating outwards to the renal cortex and centered by a contracted renal pelvis [16]. Hydronephrosis often presents with a similar pattern of hypoattenuation as the bear claw sign [16], which is a part of the diagnostic difficulty, but the mechanism leading to these similar imaging findings differs. Whereas in hydronephrosis, the hypoattenuation often represents calyceal distention, in XGP they represent infiltrating inflammation [5]. Additionally, there have been rare reports of renal enlargement from tuberculosis infection [17], further underscoring the need for a broad differential in patients with complex medical history and multiple risk factors for disease.

Although there has been a trend to treat XGP with conservative percutaneous drainage, this case reinforces the necessity to consider nephrectomy in patients with chronic disease and declining kidney function. As it is often difficult to perform a laparoscopic nephrectomy on a XGP kidney, physicians may instead elect to do a laparoscopic-assisted nephroureterectomy [18]. XGP mimics the findings of other pathological conditions, and no single clinical or radiologic feature is diagnostic of XGP. This case illustrates a young male with destructive, concurrent XGP, and UTUC. Knowing the association of XGP and UTUC can help physicians properly manage XGP by raising their awareness of associated malignancies. Moreover, this report may guide future research exploring the development and management of both conditions. Future studies analyzing the genetics of each condition, mechanisms involved in inflammatory pathways, and the effects of the urinary tract microbiome will be useful in elucidating more similarities in the development of UTUC and XGP.

## Data Availability

All data generated or analyzed during this study are included in this published article.

## Abbreviations

CT: Computed tomography
XGP: Xanthogranulomatous pyelonephritis
UC: Urothelial carcinoma
UTUC: Upper tract urothelial carcinoma
GC: Gemcitabine and cisplatin.
